# Perinatal outcome after vacuum assisted delivery with digital feedback on traction force; a randomised controlled study

**DOI:** 10.1186/s12884-021-03604-z

**Published:** 2021-02-26

**Authors:** Stefhanie Romero, Kristina Pettersson, Khurram Yousaf, Magnus Westgren, Gunilla Ajne

**Affiliations:** 1 Pregnancy Care & Delivery K57, Karolinska University Hospital, 141 86 Stockholm, Sweden; 2 Division of Obstetrics and Gynecology, Clintec, Karolinska Institutet, 141 86 Stockholm, Sweden; 3 School of Technology and Health, Royal Institute of Technology, Stockholm, Sweden

**Keywords:** Vacuum assisted delivery, Traction force, Haptic feedback, Neonatal outcome

## Abstract

**Background:**

Low and mid station vacuum assisted deliveries (VAD) are delicate manual procedures that entail a high degree of subjectivity from the operator and are associated with adverse neonatal outcome. Little has been done to improve the procedure, including the technical development, traction force and the possibility of objective documentation. We aimed to explore if a digital handle with instant haptic feedback on traction force would reduce the neonatal risk during low or mid station VAD.

**Methods:**

A two centre, randomised superiority trial at Karolinska University Hospital, Sweden, 2016–2018. Cases were randomised bedside to either a conventional or a digital handle attached to a Bird metal cup (50 mm, 80 kPa). The digital handle measured applied force including an instant notification by vibration when high levels of traction force were predicted according to a predefined algorithm. Primary outcome was a composite of hypoxic ischaemic encephalopathy, intracranial haemorrhage, seizures, death and/or subgaleal hematoma. Three hundred eighty low and mid VAD in each group were estimated to decrease primary outcome from six to 2 %.

**Results:**

After 2 years, an interim analyse was undertaken. Meeting the inclusion criteria, 567 vacuum extractions were randomized to the use of a digital handle (*n* = 296) or a conventional handle (*n* = 271). Primary outcome did not differ between the two groups: (2.7% digital handle vs 2.6% conventional handle). The incidence of primary outcome differed significantly between the two delivery wards (4% vs 0.9%, *p* < 0.05). A recalculation of power revealed that 800 cases would be needed in each group to show a decrease in primary outcome from three to 1 %. This was not feasible, and the study therefore closed.

**Conclusions:**

The incidence of primary outcome was lower than estimated and the study was underpowered. However, the difference between the two delivery wards might reflect varying degree of experience of the technical equipment. An objective documentation of the extraction procedure is an attractive alternative in respect to safety and clinical training. To demonstrate improved safety, a multicentre study is required to reach an adequate cohort. This was beyond the scope of the study.

**Trial registration:**

ClinicalTrials.gov NCT03071783, March 1, 2017, retrospectively registered.

**Supplementary Information:**

The online version contains supplementary material available at 10.1186/s12884-021-03604-z.

## Background

Vacuum assisted delivery (VAD) is performed in cases of fetal distress or labour dystocia as an alternative to obstetric forceps or caesarean section (CS) [1] and the prevalence varies greatly between countries. In Scandinavian countries it is used in 7 % of all deliveries, and arguably be an explanation of why these countries exhibit relatively lower caesarean section rates [2]. Adverse neonatal outcome seems to be associated to low and mid station VAD [3] and even though serious complications are rare, some seem to be overrepresented at VAD in comparison to spontaneous vaginal deliveries (SVD), e.g. intracranial haemorrhage (ICH) and asphyxia [4–7]. If this is due to the prolonged intrauterine forces exerted on the foetus [8, 9] or poor clinical judgement [10–12] is difficult to establish. In comparison to forceps and CS at the second stage of labour, some diagnoses are still overrepresented at VAD, e.g. subgaleal haematoma [7], but the overall increased risk of severe perinatal outcome at VAD is not consistent throughout the studies [13–16]. Why these discrepancies exist is difficult to say but it might be due to bias by indication, inconsistent information on the second stage or other aspects of the extraction procedure.

Even though technical progress is possible, also within obstetric care, little has been done to further develop the VAD procedure. A digital vacuum extraction handle has been developed by our research group at the Division of Obstetrics and Gynaecology, KI, in collaboration with the Royal Institute of Technology in Stockholm, Sweden [17]. It monitors real time traction forces and provides objective documentation on the procedure, which seems to increase adherence to existing guidelines [18]. A predictive algorithm for high level of traction force is incorporated in the handle. When the algorithm predicts a high level of traction force, from pull two and onward, it gives an instant feedback by vibration [19].

Knowledge regarding traction force is recently increasing. The previously suggested peak force limit for a metal cup to detach of 216-220 N [20–23] was shown to be clearly underestimated [17]. In addition, operators severely underestimate applied force and a high level of total traction force is associated with adverse neonatal outcome and the need of neonatal intensive care [17, 19].

The aim with the current study was to perform a randomized controlled study designed to evaluate whether the use of a digital handle during low or mid station vacuum extractions could reduce severe perinatal outcome in a two-centre setting and to test a predictive algorithm for high levels of traction force.

## Methods

### Study design

This was a prospective randomised controlled study including two delivery wards within Karolinska University Hospital, Sweden. Patients were recruited between 2016 and 2018. During the period involved, a total of 18,393 deliveries were recorded at the two delivery wards. Data collection was done from the electronic medical record system (Obstetrix®). The trial followed the CONSORT guidelines and was registered at the Swedish Medical Product Agency January 2016 and noted at ClinicalTrials.gov (ID: NCT03071783). The trial and technical device were also reviewed by the Swedish Health and Social Care Inspectorate from April 2016 until October 2016.

Patients were informed about the ongoing study through a written document upon their arrival to the delivery room and had the possibility to actively decline their participation. There were also informative posters throughout the delivery department.

### Participants

Possible eligible women for the study were all women giving birth at the two delivery wards and in the need of a vacuum assisted delivery with a gestational length ≥ 37 + 0 weeks, carrying a singleton pregnancy in cephalic position and met the national criteria for a vacuum assisted delivery [24]. Exclusion criteria were preterm delivery < 37 + 0 gestational weeks or multiple pregnancies.

### Interventions

Enrolled cases for vacuum extractions were randomised either to be undertaken with a conventional metal handle or a digital handle attached to a metal Bird cup 50 mm with 80 kPa pressure. The digital handle was developed by the Division of Obstetrics and Gynaecology, Institute of Clinical Science Intervention and Technology (CLINTEC), KI together with the Royal Institute of Technology (KTH). It was approved by the Swedish Health and Social Care Inspectorate (IVO) as a medical product used in a research setting (Dnr: 902–2016).

**Fig. 1:**
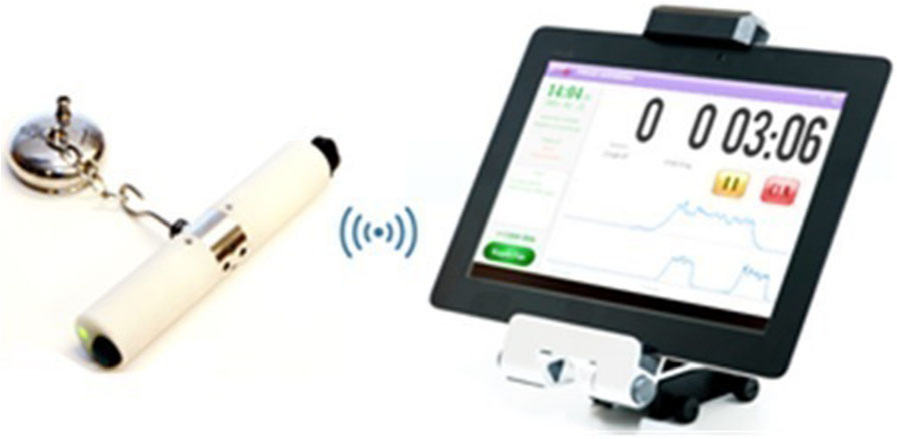
Vacuum Extraction Intelligent Handle – 3 with tablet computer

The Vacuum Extraction Intelligent Handle-3 (VEIH3) (Fig. 1) includes a regularly calibrated force sensor and instrumentation, allowing it to connect via Bluetooth® to a tablet computer for data collection. All recordings are transferred to a computer where the software MATLAB® is used to visualize, analyse and perform calculations on the force recordings. During a vacuum extraction with the VEIH3, a predictive algorithm for high level of traction force runs on the handle’s instrumentation. This algorithm is based on 277 low and mid vacuum extractions subjectively regarded as heavy by the operator [19]. If the algorithm predicts that a high level of traction force is used, a haptic feedback is provided to the operator in the form of vibration in the handle at pull two and onwards. The used prediction model for subjectively heavy vacuum extractions shows at the second pull a specificity of 0.76, a sensitivity of 0.87, a positive predictive value (PPV) of 0.56 and a negative predictive value (NPV) of 0.94, and at the third pull a specificity of 0.87, a sensitivity of 0.70, a PPV of 0.65 and a NPV of 0.89.

Previous studies with the digital handle were carried out at delivery ward B between 2013 and 2015, but without haptic feedback response. Both wards were introduced to the haptic feedback response and its clinical background, including NPV and PPV, 2 months prior to the start of the study. No recommendations on how to act on this information was given, since there was yet no proof of its effect, and the decision how to deliver depends on the clinical situation. The staff was informed that haptic feedback was associated with an increased risk for a difficult extraction. Two assistant nurses and two obstetricians at each delivery ward acted as coordinators to assist on daily basis during the clinical investigation. Four devices, two for each investigational site, were marked with a unique lot number and labelled as “Exclusively for use in clinical investigation”. No part of the digital handle or its equipment was ever in direct contact with the patient or the fetal head.

During the 2 years, there was a continuous maintenance of the four devices, including calibrations according to protocol, but the force recordings were never needed recalibration. The plastic shells of the handle were replaced in three out of the four digital handles due to cracking.

There were no changes neither in clinical care during delivery nor in the indications for a vacuum assisted delivery. The fetal head station was assessed according to international guidelines [25], defining *mid* station when the fetal head was above level plus 2, but not above the ischial spines, *low* station at level plus 2 or more, but not on the pelvic floor, and *outlet* station when the leading part had reached the pelvic floor. Documentation was carried out according to the national standard protocol of vacuum assisted delivery, used since 1992 in Sweden, including a parameter where the obstetrician evaluates the extraction procedure as “easy”, “average” or “heavy”.

### Outcomes and monitoring

Outcomes were calculated for low and mid station vacuum extractions, excluding outlet station according to study protocol. The primary outcome was specified as a composite of perinatal hypoxic ischaemic encephalopathy (HIE), intracranial haemorrhage (ICH), death, seizures and subgaleal hematoma. Collected neonatal characteristics and secondary neonatal outcomes were the individual components of the primary outcome, along with admission to a neonatal intensive care unit, Apgar score less than 7 at 5 minutes, pH in the umbilical artery less than 7.10 or 7.00, brachial plexus injury (Erb’s palsy), fractures, cephalohematoma, hyperbilirubinemia, birthweight and gender. Collected maternal characteristics and secondary outcomes were age, parity, weight, height, duration of first and second stage of labour until indication for vacuum extraction, station and position of fetal head and indication to perform a vacuum assisted delivery. Vacuum procedural outcomes were duration of extraction, number of pulls, cup detachment, number of cup detachments and failed extraction. When the digital handle was used, peak traction force, total traction force and haptic feedback were noted and included in per protocol subgroup analyses.

The principal investigator together with the responsible researchers at each delivery ward undertook standard assessment of safety, with reporting of severe adverse events and adverse events following procedures stated by the Swedish Health and Social Care Inspectorate (D:nr 902–2016). An interim analysis was planned after 1 year.

### Sample size

The sample size calculation was based on an incidence of 7 % of HIE among heavy low and mid station VAD and 0.8% in non-heavy low and mid VAD in Pettersson et al. 2015 [17]. A calculated sample size of 380 low and mid station vacuum extractions cases and controls respectively was estimated to lower the incidence of a composite of primary outcome (HIE, ICH, subgaleal haematoma, seizure and death) among all low and mid VAD from six to 2 % with 80% power and 0.05% significance level (superiority testing).

### Randomisation

When a decision was made to perform a vacuum assisted delivery and all inclusion criteria were fulfilled, the computerised randomisation program running on the tablet computer of the VEIH3 in the delivery room was initiated by the assistant nurse. Each tablet computer was mounted on a vacuum pump and connected to a specific digital handle (VEIH3 1–4). A true randomisation was enabled in the software to aim for a 1:1 allocation to each trail group. After completing the randomisation and allocating the patient to one of the two study groups, the assistant nurse handed the appointed handle (either the digital or the conventional handle) to the operator. The operator was not involved in the randomisation process.

### Data collection

When the study was closed, force measurement recorded data was retrieved from the tablet computers and analysed using MATLAB® by the technical engineer K Yousaf and Dr. S Romero. Traction force data was linked to the clinical data using the unique personal identification number allocated to each person in Sweden. Clinical data was prospectively entered in standardised electronic medical records (Obstetrix®) by midwives and clinicians during pregnancy, delivery and postpartum. Data collection of clinical variables was carried out by Dr. S Romero. To estimate selection bias we compared the baseline characteristics and pregnancy outcomes of the study participants with the non-randomised patients who underwent a vacuum extraction delivery at the two delivery wards during the study period.

### Statistical analyses

Main analyses were performed on the intention to treat population. The analysis and presentation followed the recommendation of the CONSORT group. The primary statistical analysis was to estimate the incidence of primary outcome in the group using the digital handle compared to the group using the conventional handle, with a Fisher’s exact test at a significance level of 0.05. When comparing the secondary outcomes, a Fisher’s exact test was used for the categorical variables (with numbers and percentages), a Student’s T-test for continuous variables with a standard distribution (with means and standard deviations) and a Wilcoxon rank-sum test for the continuous variables with a non-standard distribution (with medians and min-max range). Prespecified subgroup analysis was done for the perinatal outcome diagnosis, comparison between the two delivery wards, and traction force data for cases delivered with the digital handle. A complementary analysis was performed in order to compare the primary perinatal composite outcome on the per protocol population. Statistica© (StatSoft Inc., Tulsa, OK, USA) was used for univariate statistical analysis of all data.

### Patient and public involvement

Pregnant women were not involved in the design, outcome measures or recruiting plans of the study. Likewise, they were not invited to give advice on interpretation of results. The results will be available to the public through a popular science article.

## Results

**Fig. 2:**
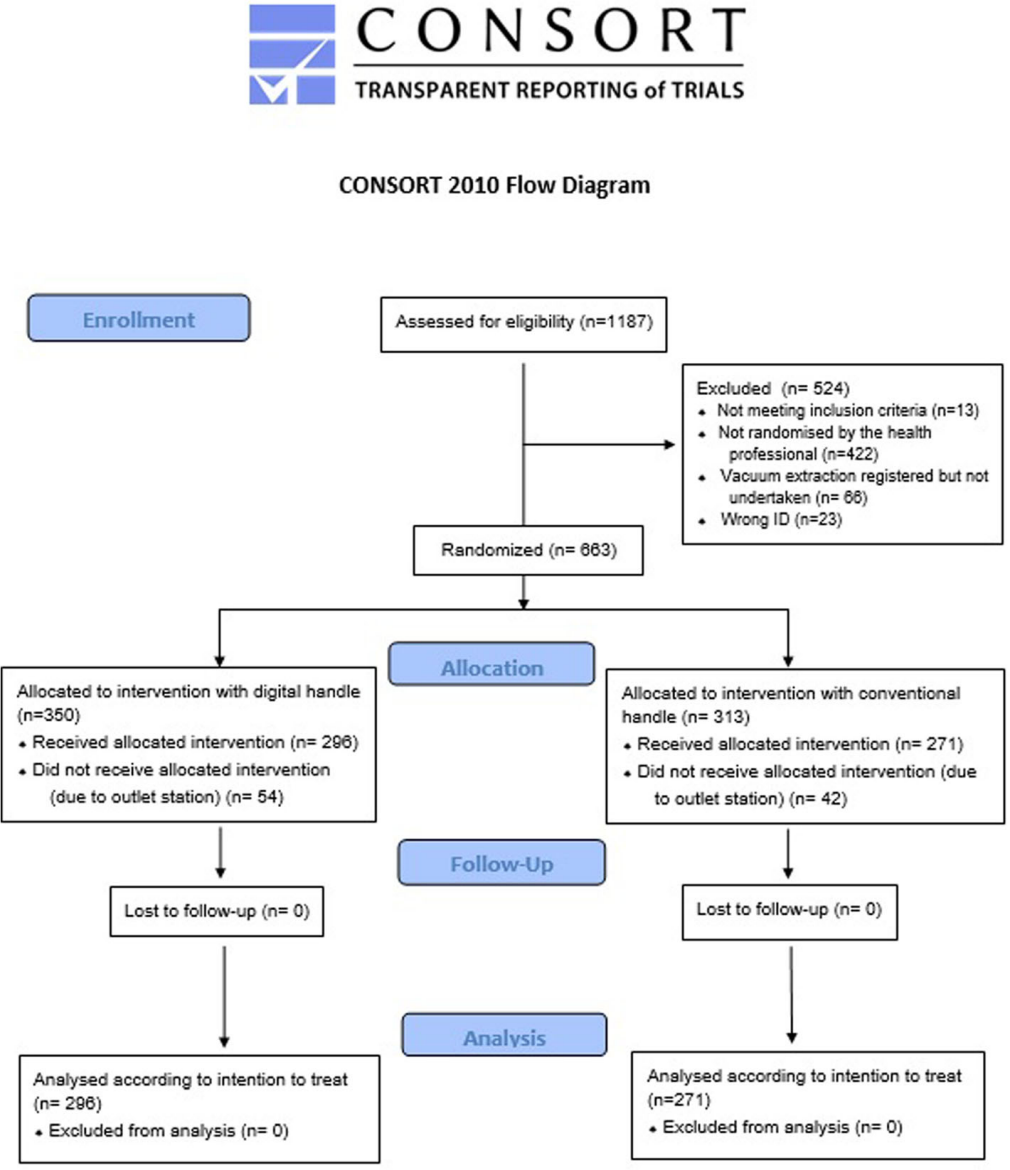
CONSORT flow diagram of eligibility, randomisation, allocation and analysis. Available at www.consort-statement.org/consort-statement/flow-diagram

An interims analysis was planned after 1 year, but due to fewer included cases than expected it was postponed until June 2018. In June 2018, 765 cases were registered and after exclusions, 663 were randomized to a digital or a conventional handle. Eligible cases included in the intention to treat analysis are described in Fig. 2. After exclusion of outlet station vacuum extractions, 567 low and mid station vacuum extractions were analysed (296 in the intervention digital handle group and 271 in the conventional handle group (Fig. 2). There was no significant difference in primary outcome between the two study groups. The incidence of primary outcome was lower than estimated, 2.6% in comparison to the 6 % expected. Table 1 compares randomised and non-randomised vacuum extractions. The indication for vacuum extraction was more often dystocia, and more extractions were undertaken with fetal head at low station, in the randomised group. The primary outcome did not differ (Table 1).

**Table 1:** Analysis between randomized and non-randomized VAD

	Non randomised (***n*** = 422) n (%)	Randomised (***n*** = 663) n (%)	*p*-value
**Primary outcome** (HIE, ICH, seizure, death, subgaleal hematoma)	13 (3%)	16 (2.4%)	0.56
**Maternal characteristics**			
Age^1^	31±5	31±5	0.68
BMI (kg/m^2^)^1^	23±4	24±4	0.47
Missing	20 (5%)	26 (4%)	
Nulliparous	312 (74%)	521 (79%)	0.09
Gest. length, days¹	283±8	283±8	0.74
**Characteristics: delivery and vacuum extraction**			
Time cx 3 cm – fully dilated, hours¹	8±5	9±5	0.50
Time fully dilated to vacuum extraction, hours¹	2±1.5	3±1.5	0.30
Indication			
- OFHR	198 (47%)	207 (31%)	<0.001
Station			
- Mid	189 (45%)	306 (46%)	0.66
- Low	135 (32%)	261 (39%)	<0.05
- Outlet	78 (18%)	96 (14%)	0.09
Number of pulls, n^2^	2 (4-2)	2 (4-2)	0.61
Missing	14 (3%)	15 (2%)	
Vacuum extraction duration, min^2^	5.3 (8.3-3.0)	4.0 (8.0-4.0)	0.73
Position OAP	369 (87%)	596 (90%)	0.23
Failed vacuum extraction	33 (8%)	56 (8%)	0.74
Pop-off	27 (6%)	51 (8%)	0.47
Pop-off ≥2	7 (2%)	9 (1%)	0.79
**Perinatal characteristics**			
Birth weight, g¹	3540±489	3558±464	0.40
NICU	41 (10%)	61 (9%)	0.83
Gender			0.74
- Male	242 (57%)	386 (58%)	
- Female	180 (43%)	277 (42%)	
pH<7.00	7 (2%)	11 (2%)	1
Missing	64 (15%)	65 (10%)	
pH<7.10	54 (13%)	84 (13%)	0.70
Missing	64 (15%)	65 (10%)	
APGAR<7 at 5 min	15 (4%)	19 (3%)	0.59
Fracture*	5 (1%)	6 (1%)	0.76
Plexus injury	4 (1%)	5 (0.8%)	0.74

*VAD* vacuum assisted delivery, *ICH* intracranial hemorrhage, *HIE* hypoxic ischemic encephalopathy, *BMI* body mass index, *OFHR* ominous fetal heart rate, *OAP* occipital-anterior position. mean±sd¹, IQR (Q3-Q1)^2^, *clavicle, skull, humerus

### Primary outcome

Primary outcome was a composite of ICH, HIE, subgaleal hematoma, seizures and neonatal death. The primary outcome occurred in 2.7% (8/296) in the intervention group and 2.6% (7/271) in the conventional handle group (*p* = 1.00). No neonatal death occurred. Subgaleal hematoma was the main contributor to primary outcome: 1.6% (9/567). HIE accounted for 0.7% (4/567) of the cases, ICH for 0.4% (2/567) and seizures for 0.7% (4/567).

### Secondary outcomes

Table 2 shows the secondary outcomes. A significant shorter extraction time was noted in the group using the conventional handle. Also, a tendency towards more male foetuses was seen in the intervention group, but this was not statistically significant. Apart from this there were no other differences between the groups.

**Table 2:** Clinical characteristics and secondary outcomes in the randomized cohort (by intention to treat), low and mid station VAD

	DH (***n*** = 296) n (%)	CH (***n*** = 271) n (%)	*p*-value
**Maternal characteristics**			
BMI (kg/m^2^)¹	24±4	24±4	0.48
Missing	16 (5%)	9 (3%)	
Nulliparous	233 (79%)	205 (76%)	0.42
Gest. length, days¹	283±9	282±8	0.25
Age¹	31±5	32±5	0.63
Height¹	164±7	164±7	0.68
**Characteristics: delivery and vacuum extraction**			
Failed vacuum extraction	25 (8%)	31 (11%)	0.26
Time fully dilated to vacuum extraction, hours¹	3.1±1.5	2.9±1.5	0.08
Time cx 3 cm – fully dilated, hours¹	8.8±5	8.9±5	0.76
Subjective heavy extraction	53 (18%)	51 (19%)	0.83
Missing	12 (4%)	8 (3%)	
Epidural	248 (84%)	223 (82%)	0.66
Oxytocin	285 (96%)	260 (96%)	0.83
Indication OFHR	94 (32%)	79 (29%)	0.52
Station			
- Mid	156 (53%)	150 (55%)	0.56
- Low	140 (47%)	121 (45%)	0.56
Number of pulls, n^3^	2 (4-2)	2 (4-2)	0.67
Missing	7 (2%)	7 (3%)	
Vacuum extraction duration, min^3^	6 (9-3)	5 (9-4)	0.61
Position (OAP)	273 (92%)	237 (87%)	0.07
Pop-off	23 (8%)	24 (9%)	0.65
Pop-off ≥2	3 (1%)	5 (2%)	0.49
Shoulder dystocia	4 (1%)	2 (0.7%)	0.69
Perinatal characteristics			
Birth weight, g¹	3570±490	3569±423	0.98
NICU	25 (8%)	27 (10%)	0.56
NICU, days²	2 (1-25)	2 (1-17)	0.99
Gender			0.06
- Male	187 (63%)	150 (55%)	
- Female	109 (37%)	131 (45%)	
pH<7.00	5 (2%)	2 (0.7%)	0.45
Missing	30 (9%)	27 (11%)	
pH<7.10	37 (13%)	33 (12%)	1
Missing	30 (9%)	27 (11%)	
APG<7 at 5 min	8 (3%)	10 (4%)	0.63
Plexus injury	1 (0.3%)	3 (1%)	0.35
Cefalohematoma	40 (14%)	28 (10%)	0.30
Hyperbilirubinemia	27 (9%)	17 (6%)	0.21
Fracture	1 (0.3%)	3 (1%)	0.35

*VAD* vacuum assisted delivery, *DH* digital handle, *CH* conventional handle, *BMI* body mass index, *Cx* cervix, *OFHR* ominous fetal heart rate, *OAP* occipital-anterior position, *APG* Apgar, *NICU* neonatal intensive care unit. mean±sd¹, median (min-max)², IQR (Q3-Q1)^3^, *clavicle, skull, humerus

### Per protocol analyses

The pre-specified analyses of the per protocol population included 246 women in the group with the digital handle and 321 women in the group with the conventional handle. Fifty cases (17%) had a violation of protocol in the group with the digital handle. The reasons for protocol violations were rerandomization in 48 cases (the randomization button was pressed more than once when preparing the vacuum extraction equipment) and two cases with delayed force recordings making the force recording data incomplete. Reasons for protocol violation is referred to insufficient knowledge of the equipment and not technical instability. The primary perinatal outcome was recorded in six cases in the group with the digital handle (2.4%) and nine in the group with the conventional handle (2.8%) (*p* = 1.00). Supplementary Table S1 shows the results of the secondary outcomes.

### Subgroup analyses

Subgroup analyses comparing cases with primary outcome versus cases without primary outcome showed a significant increased incidence of vacuum extraction procedure variables linked to a high level of traction force, such as increased number of pulls, vacuum extraction attempts followed by caesarean section or forceps or pop-offs (Table 3).

**Table 3:** Results comparing variables in the group with primary outcome with the group without primary outcome (mid + low vacuum assisted deliveries)

	Primary outcome (***n*** = 15)	Not primary outcome (***n*** = 552)	*p*-value
Indication dystocia	10 (67%)	384 (70%)	0.78
Pop-offs	5 (33%)	42 (8%)	<0.05
Converted vacuum extraction	5 (33%)	51 (10%)	<0.05
Nº pulls (median (min-max))	4 (2-10)	3 (1-12)	<0.05
Vacuum extraction duration (median (min-max))	9 (3-21)	6 (1-26)	<0.05
Station mid	11 (73%)	295 (53%)	0.08

When comparing the results between the two delivery wards a statistically significant difference in primary outcome was found with an incidence of 4% at delivery ward A vs 0.9% at delivery ward B (*p* < 0.05).

Per protocol subgroup analysis by haptic feedback response (vibration) by the digital handle is summarized in Table S2. It shows that 34% (83/246) had a haptic feedback response, and all six cases with primary outcome. Out of these, one was delivered at ward B (converted to CS) and five at ward A (two converted to CS and three delivered vaginally after several consecutive pulls).

Out of the vacuum extractions in the digital handle group, 18% were subjectively evaluated as heavy (42/234). Recalculation of the predictive model at pull three showed that the sensitivity was unchanged (0.70 vs 0.71), but the specificity decreased (0.87 vs 0.74). The prevalence of heavy extractions was lower in the present study compared to the cohort included when creating the algorithm (18% vs 26%), significantly decreasing the PPV from 0.64 to 0.36. The NPV increased from 0.89 to 0.93.

Median peak traction force was 181 N (42–419) and median total traction force was 187 N minutes (14–1564). Table 4 shows the peak force and total force for the cases with primary outcome in comparison with those without.

**Table 4:** Traction forces by protocol comparing cases with primary outcome and those without primary outcome

	Primary outcome (***n*** = 6)	Not primary outcome (***n*** = 236)	*p*-value
**Peak force** (N)¹	220 ± 35	183 ± 56	0.11
**Total force** (N minutes)²	460 (255-833)	182 (14-1565)	<0.01

¹mean ±SD, ²median (min-max)

## Discussion

### Main findings

In this randomized control study we compared the primary outcome of severe neonatal diagnosis between a group randomised to the use of a digital handle during low or mid station vacuum extraction procedure (giving an instant haptic feedback on high traction force) and a group with a conventional vacuum extraction handle. No statistically significant difference was seen in primary perinatal outcome. The overall incidence of primary perinatal outcome was 2.6% and differed significantly between the two participating delivery wards (4% vs 0.9%). Cases with primary outcome had evidence of high level of traction force used in the vacuum extractions. Technical monitoring was stable.

### Strengths and weaknesses

Even though the two included delivery wards are organised within the same hospital with analogous guidelines, skills and team training, a great difference in primary outcome was noted. The outcome after a vacuum extraction depends on a multi-factorial procedure from indication, maternal and fetal variables to professional skills. Ward A had used the digital handle 2 months prior to this study for training purposes, while ward B had used the handle during 3 years prior to this study on a voluntary basis in clinical observational vacuum traction force studies without a haptic feedback [17]. A limitation with the current study is the lack of strict clinical recommendations when the haptic alarm notified the operator. The reason for this was that the research group had limited information on outcome and that the algorithm only predicted a subjective variable – difficult extraction.

Cases with primary neonatal outcome had objective parameters linked to heavy or difficult extractions. Awareness and training of objective parameters, including traction force, is one way to improve neonatal outcome in low and mid station vacuum extraction. If haptic feedback on traction force is an objective parameter to reach this in clinical settings or as an educational tool can only be speculated at the moment.

The incidence of primary outcome was lower than estimated and the calculated sample size not sufficient enough for a superiority intention to treat analysis. A sample size of approximate 800 cases of low or mid station vacuum extractions in each group will be needed in a future multicentre study to test a superiority hypothesis from three to 1 % incidence of primary outcome in each group, or 1500 cases in each group for a 50% reduction.

Out of all the vacuum extractions, 64% were randomised and included in the study. The distribution of randomised cases to the two study groups was equal. In non-randomised cases, ominous fetal heart rate was a more common indication for the vacuum extraction procedure than in the randomised cases. An explanation to this might be a lower propensity to randomise a case to a study in such a stressful situation. Likewise, there were fewer cases with fetal vertex at low station among non-randomised cases, but mid station did not differ and there was a tendency of more outlet station in the non-randomised group. The response analyses showed otherwise an equal distribution, including incidence of primary outcome.

Technical monitoring was stable, but knowledge about the equipment was not sufficient enough throughout the whole study time period. The possibility to re-randomize the same patient was the most common reason for violating the study protocol.

The predictive algorithm for heavy low or mid station vacuum extraction was tested in a clinical setting. The relatively high NPV in the tested algorithm may be a parameter of reassurance regarding limited effect by traction force on the foetus during the extraction.

### Interpretation

The overall risk for severe adverse perinatal outcome with vacuum extraction seems to be associated mainly to low and mid station extractions and is higher when compared to caesarean section [6, 15, 26]. The causality is difficult to establish since many factors are involved. A recent study suggests that the duration of the extraction procedure may be closer associated with a severe perinatal outcome than other extraction characteristics [27]. Considering maternal risks, the incidence of maternal morbidity and mortality seems to be lower after a vacuum extraction procedure in comparison to caesarean section at a second stage of labour [26].

We know from other studies that it is possible to apply higher traction forces than earlier believed possible when using a metal Bird cup (50 mm, 80 kPa) in term pregnancies. The vacuum counterforce is not self-limiting at 216 Newtons, which was earlier suggested to be a force limit [20]. So, a question rises if high traction force can put the foetus at risk for intracerebral injury that we are not aware of. Hypothetically, this could be forces affecting intracerebral perfusion pressure or significant moulding of soft and hard fetal head tissue and risk of tearing. The predictive algorithm used is based on a subjective perception of what a high level of traction force is, but we can assume that the algorithm is giving haptic feedback at an objectively high level of traction force, as results from previous studies have shown that applied force during a subjective high level of traction force is far higher than subjectively estimated.

All cases with primary neonatal outcome delivered with the technical device gave a haptic feedback response to the operator during the procedure. As described in the method section, the study protocol did not suggest any guideline on what to do if haptic feedback response was given but was up to the operator to decide. Obviously, the clinician involved handled this information very differently, as primary outcome with digital handle was unevenly distributed between the wards. One possible alternative would have been to stop the vacuum extraction and convert to a caesarean section. If this alternative would decrease the risk for severe perinatal outcome was not tested in the present study. It is known though that a failed vacuum extraction is a risk factor for adverse perinatal outcome [28] and further risk seems to occur when multiple modes are used [29, 30]. In this study, 11 % were converted to another delivery mode; 0.8% to forceps and 10 % to caesarean section.

If vacuum extraction attempt with early conversion can lower the risk remains an open question. Mid high extractions with a metal cup are an increasingly rare obstetrical procedure, which is not practiced in all countries. In the Scandinavian countries, and in some other European countries with rather low caesarean section rates, it is still part of the clinical obstetrical arsenal. How much this reflects on the caesarean section rate is unknown. We believe that, if this clinical practice should prevail, it needs to be performed with a high degree of safety. One way of identifying risks and making an intervention safer is by objective monitoring.

The fact that the labour ward where the technical device was used 3 years prior to the start of the study had significant lower incidence of primary outcome, raises the question if perception of traction force and assessment of the extraction progress are skills that can be practiced? The stable technical monitoring from the device creates an opportunity to objectively document the vacuum extraction procedure. In cases with severe perinatal outcome, this documentation may give the evaluator new objective clinical data to better assess the situation.

The results from the present study will affect future plans on a multicentre study if addressing the same primary question. The technical setting is easily corrected to disable rerandomization. Sample size will require multicentre approach and interindividual skill differences will probably affect the result. We believe that the individual based randomization is superior to cluster randomization considering the different outcome in the two delivery wards.

## Conclusion

The incidence of primary outcome was lower than estimated and the study was underpowered to show an effect in reducing severe adverse neonatal outcome. The difference between the two delivery wards might reflect varying degree of experience of the technical equipment. Digital objective documentation might increase the compliance to guidelines and is an attractive alternative in respect to safety and clinical training. Perception and assessment to traction force progress are skills that are possible to practice using digital educational models with continuous feedback.

To demonstrate an improved safety, a multicentre study is required to reach an adequate cohort, but this was beyond the scope of the study.

## Supplementary Information

**Additional file 1: Table S1.** Results analysis secondary outcome (by protocol) (low- and mid vacuum extractions).

**Additional file 2: Table S2.** Per protocol subgroup analysis of haptic feedback response and conversion of VAD.

**Additional file 3.** Clinical Protocol Vacuum Extraction.

## Data Availability

Data are available upon reasonable request and should be addressed to Stefhanie.romero@sll.se. Non-identified individual participant data will be available to medical researchers by request in accordance with local registration and ethical approval, when the article has been published, and ten years ahead. All proposals requesting data access will need to specify an analysis plan and will need approval of the scientific board before any data can be released.

## Abbreviations

RCT: Randomized controlled trial
HIE: Hypoxic ischaemic encephalopathy
IC H: intracranial haemorrhage
DH: Digital handle
CH: Conventional handle
PPV: Positive predictive value
NPV: Negative predictive value
