# Supplementary material for: Perinatal outcome after vacuum assisted delivery with digital feedback on traction force; a randomised controlled study
Source: BMC Pregnancy Childbirth. 2021 Feb 26;21:165. doi: 10.1186/s12884-021-03604-z (PMC7913459; doi:10.1186/s12884-021-03604-z)
Supplement: Supplementary file 1 — Additional file 1: Table S1. Results analysis secondary outcome (by protocol) (low- and mid vacuum extractions). [file 12884_2021_3604_MOESM1_ESM.docx]

| Table S1  Results analysis secondary outcome (by protocol) (low- and mid vacuum extractions) | | | | | | |
| --- | --- | --- | --- | --- | --- | --- |
|  | **DH (n=246)**  **n (%)** | | **CH (n=321)**  **n (%)** | | p-value | |
| Maternal characteristics | | | | | | |
| BMI (kg/m^2^)¹  Missing | | 24±4  13 (5%) | | 24±4  12 (4%) | | 0.99 |
| Nulliparous | | 192 (78%) | | 246 (77%) | | 0.76 |
| Gest. length, days¹ | | 282±8 | | 283±9 | | 0.12 |
| Age¹ | | 32±5 | | 31±5 | | 0.63 |
| Hight¹ | | 164±7 | | 164±7 | | 0.97 |
| Characteristics: delivery and vacuum extraction | | | | | | |
| Failed VE | | 22 (9%) | | 34 (11%) | | 0.57 |
| Time fully dilated to vacuum extraction, hours¹ | | 3.1±1.5 | | 2.8±1.5 | | <0.05 |
| Time cx 3 cm – fully dilated, hours¹ | | 9±5 | | 9±5 | | 0.72 |
| Subjective heavy extraction  Missing | | 42 (17%)  12 (5%) | | 62 (19%)  8 (2%) | | 0.66 |
| Epidural | | 208 (85%) | | 263 (82%) | | 0.43 |
| Oxytocin | | 239 (97%) | | 306 (95%) | | 0.28 |
| Indication OFHR | | 80 (33%) | | 93 (29%) | | 0.41 |
| Station   - Mid - Low | | 127 (52%)  119 (48%) | | 179 (56%)  142 (44%) | | 0.35 |
| Number of pulls, n^3^  Missing | | 2 (4-2)  6 (2%) | | 2.5 (4.5-2)  8 (2.5%) | | 0.39 |
| Vacuum extraction duration, min^3^ | | 5 (8-3) | | 5 (9-4) | | 0.34 |
| Position (OAP) | | 227 (92%) | | 283 (88%) | | 0.12 |
| Pop-off | | 21 (9%) | | 26 (8%) | | 0.88 |
| Pop-off ≥2 | | 2 (0.8%) | | 6 (2%) | | 0.48 |
| Shoulder dystocia | | 3 (1%) | | 3 (1%) | | 1 |
| Perinatal characteristics | | | | | | |
| Birth weight, g¹ | | 3580±444 | | 3557±478 | | 0.56 |
| NICU | | 21 (9%) | | 31 (10%) | | 0.66 |
| NICU, days² | | 2 (1-25) | | 2 (1-17) | | 0.99 |
| Gender   - Male - Female | | 160 (65%)  86 (35%) | | 177 (55%)  144 (45%) | | <0.05 |
| pH<7.00  Missing | | 5 (2%)  22 (9%) | | 2 (0.6%)  35 (11%) | | 0.25 |
| pH<7.10  Missing | | 30 (12%)  22 (9%) | | 40 (13%)  35 (11%) | | 0.90 |
| APG<7 at 5 min | | 6 (2%) | | 12 (4%) | | 0.47 |
| Cefalohematoma | | 32 (13%) | | 36 (11%) | | 0.52 |
| Hyperbilirubinemia | | 18 (7%) | | 26 (8%) | | 0.75 |
| Fracture^*^ | | 1 (0.4%) | | 3 (0.9%) | | 0.64 |
| DH: digital handle, CH: conventional handle, BMI: body mass index, Cx: cervix, OFHR: omnious fetal heart rate, OAP: occipital-anterior position, APG: Apgar, NICU: neonatal intensive care unit. mean±sd¹, median (min-max)², IQR (Q3-Q1)^3^, ^*^clavicle, skull, humerus | | | | | | |
