# Supplementary material for: Perinatal outcome after vacuum assisted delivery with digital feedback on traction force; a randomised controlled study
Source: BMC Pregnancy Childbirth. 2021 Feb 26;21:165. doi: 10.1186/s12884-021-03604-z (PMC7913459; doi:10.1186/s12884-021-03604-z)
Supplement: Supplementary file 2 — Additional file 2: Table S2. Per protocol subgroup analysis of haptic feedback response and conversion of VAD. [file 12884_2021_3604_MOESM2_ESM.docx]

| Table S2  Per protocol subgroup analysis of haptic feedback response and conversion of VAD | | |
| --- | --- | --- |
|  | **DH (n= 246)** | **CH (n=321)** |
| **Converted VAD** | 25 (10%) | 31 (9.7%) |
| **Converted VAD to CS** | 22/25 (88%) | 29/31 (94%) |
| **Primary outcome and converted to CS** | 3/6 (50%) | 2/7 (29%) |
| **Subjectively heavy extraction**  **Missing** | 42/234 (18%)  12 (5%) | 62/313 (20%)  8 (2.5%) |
| **Primary outcome and subjectively heavy** | 6/6 (100%) | 2/7 (29%) |
| **Primary outcome with haptic feedback** | 6/6 (100%) | - |
| **Haptic feedback overall** | 83 (34%) | - |
| **Subjectively heavy with haptic feedback** | 30/42 (71%) | - |
| **Haptic feedback pull 3, delivered within next two contractions** | 35/54 (65%) | - |
| **Haptic feedback and converted to CS** | 12/83 (15%) | - |
| VAD: vacuum assisted delivery, DH: digital handle, CH: conventional handle, VAD: vacuum assisted delivery, CS: caesarean section | | |
