# Supplementary material for: Perinatal outcome after vacuum assisted delivery with digital feedback on traction force; a randomised controlled study
Source: BMC Pregnancy Childbirth. 2021 Feb 26;21:165. doi: 10.1186/s12884-021-03604-z (PMC7913459; doi:10.1186/s12884-021-03604-z)
Supplement: Supplementary file 3 — Additional file 3. Clinical Protocol Vacuum Extraction. [file 12884_2021_3604_MOESM3_ESM.pdf]

VE-protokoll

Kontrollera att trycket går att sänka till 80 kPa innan slangen kopplas till sugkoppen!

Pat.ID/Namn/Rum_______________________________

Time-out leds av operatören:

1. Teamdeltagare presenterar sig
2. Operatör anger indikation, förväntad svårighet (YP + VU)
3. Urinblåsa tappad? PVK, bas?
4. Smärtlindring?
5. Ev Oxytocinstimulering, läkare styr ordination av dropphöjning
6. CTG-registrering och tolkning. CTG ses bäst på dataskärmen
7. Perinealskydd, vem utför?

Läkare _____________________________ Barnmorska 1 ________________________

USK _________________________________ Barnmorska 2 ________________________

Barnläkare __________________________

VE anlagd kl.__________________+ starta tidtagning

Trycket sänkt till 80 kPa kl. _______________
Om tid medges sänk ev först till 20 kPa och kontrollera att vaginalslemhinna inte fastnat. Sänk därefter till 80 kPa.

Tidpunkt enligt tidtagaruret:

Drag 1___________Drag 2___________ Drag 3_________**OBS! Säg högt antal drag**

Drag 4___________Drag 5___________ Drag 6_________**OBS! Säg högt antal drag**

____

KLOCKSLÄPP vid drag nr :______________________________________

OBS! Uppge när 10 min, 15 min resp. 20 min har gått från det att klockan lades an.

KLOCKA AV (tid): ______________________________

HUVUD UTE (tid): ______________________________

PARTUS(tid): __________________________________

Avslutning: summering förlopp, ansvar bristning
Dokumentera i VE-mall att time-out utförts, tidpunkt för ev klocksläpp, klockposition på fosterhuvudet, perinealskydd utfört hur och av vem.

PROTOCOL VACUUM EXTRACTION

Check that the pressure can be reduced to 80 kPa before connecting the tube to the cup!

Patient ID/Name/Delivery room ______________________________

Time-out is managed by the operator:
1. Team members introduce themselves
2. Operator indicates indication, expected difficulty (Uterus Palpation + Vaginal Exam)
3. Bladder empty? Venous access, blood group antibody screen test?
4. Analgesia?
5. Possible Oxytocin Stimulation, the doctor decide level of infusion
6. Fetal Cardio Toco Gram registration and interpretation. CTG is best seen on the computer screen
7. Perineal protection, who performs?

Doctor ____________________________ Midwife 1 ________________________

Assitant nurse ______________________ Midwife 2 ________________________

Pediatrician _________________________

Cup on, time.__________________+ start the timekeeping

If time permits, first lower to 20 kPa and check that the vaginal mucosa is not stuck. Then lower to 80 kPa.

Time according to the timer clock:

Drag 1__________Drag 2__________ Drag 3________N.B! Speak out number of pulls

Drag 4__________Drag 5__________ Drag 6________ N.B! Speak out number of pulls

POP-OFF at pull number: _____________________________________

N.B.! State when 10 min, 15 min and 20 minutes have passed since the cup was applied.

CUP OFF (time): ______________________________

HEAD OUT (time): ______________________________

PARTUS (time): __________________________________

Conclusion: summary process, responsibility failure
Document in the Vacuum Extraction template that time-out was performed, time for POP-off if applicable, cup position on the fetal head, perineal protection performed how and by whom.
